# Effector mechanisms of interleukin-17 in collagen-induced arthritis in the absence of interferon-γ and counteraction by interferon-γ

**DOI:** 10.1186/ar2787

**Published:** 2009-08-17

**Authors:** Hilde Kelchtermans, Evelien Schurgers, Lies Geboes, Tania Mitera, Jo Van Damme, Jacques Van Snick, Catherine Uyttenhove, Patrick Matthys

**Affiliations:** 1 Laboratories of Immunobiology, Rega Institute, Faculty of Medicine, Katholieke Universiteit Leuven, Minderbroedersstraat 10, B-3000 Leuven, Belgium; 2 Molecular Immunology, Rega Institute, Faculty of Medicine, Katholieke Universiteit Leuven, Minderbroedersstraat 10, B-3000 Leuven, Belgium; 3 Ludwig Institute for Cancer Research, Brussels Branch, Cellular Genetics and Experimental Units, Christian de Duve Institute of Cellular Pathology, Université Catholique de Louvain, Avenue Hippocrate 75, B-1200 Brussels, Belgium

## Abstract

**Introduction:**

Interleukin (IL)-17 is a pro-inflammatory cytokine in rheumatoid arthritis (RA) and collagen-induced arthritis (CIA). Since interferon (IFN)-γ inhibits Th17 cell development, IFN-γ receptor knockout (IFN-γR KO) mice develop CIA more readily. We took advantage of this model to analyse the mechanisms of action of IL-17 in arthritis. The role of IFN-γ on the effector mechanisms of IL-17 in an *in vitro *system was also investigated.

**Methods:**

IFN-γR KO mice induced for CIA were treated with anti-IL-17 or control antibody. The collagen type II (CII)-specific humoral and cellular autoimmune responses, myelopoiesis, osteoclastogenesis, and systemic cytokine production were determined. Mouse embryo fibroblasts (MEF) were stimulated with IL-17, tumor necrosis factor (TNF)-α and the expression of cytokines and chemokines were determined.

**Results:**

A preventive anti-IL-17 antibody treatment inhibited CIA in IFNγR KO mice. In the joints of anti-IL-17-treated mice, neutrophil influx and bone destruction were absent. Treatment reduced the cellular autoimmune response as well as the splenic expansion of CD11b^+ ^cells, and production of myelopoietic cytokines such as granulocyte macrophage colony-stimulating factor (GM-CSF) and IL-6. IL-17 and TNF-α synergistically induced granulocyte chemotactic protein-2 (GCP-2), IL-6 and receptor activator of NFκB ligand (RANKL) in MEF. This induction was profoundly inhibited by IFN-γ in a STAT-1 (signal transducer and activator of transcription-1)-dependent way.

**Conclusions:**

In the absence of IFN-γ, IL-17 mediates its pro-inflammatory effects mainly through stimulatory effects on granulopoiesis, neutrophil infiltration and bone destruction. *In vitro *IFN-γ profoundly inhibits the effector function of IL-17. Thus, aside from the well-known inhibition of the development of Th17 cells by IFN-γ, this may be an additional mechanism through which IFN-γ attenuates autoimmune diseases.

## Introduction

IL-17 is a pro-inflammatory cytokine produced by activated CD4^+ ^T cells distinct from Th1 or Th2 cells, designated as Th17 cells [1-3]. IL-17 promotes inflammation by enhancing production of cytokines such as IL-1β, TNF-α, IL-6 and receptor activator of nuclear factor-κB ligand (RANKL), as well as chemokines such as macrophage inflammatory protein (MIP)-2 and IL-8 [4-6]. Factors that promote Th17 cell differentiation and/or expansion are transforming growth factor (TGF)-β, IL-6 and IL-23 [7]. Interferon (IFN)-γ, as a contrast, strongly inhibits development of Th17 cells both *in vitro *and *in vivo *[1,3,8]. Anti-IFN-γ added during *in vitro *Th17 differentiation causes increased IL-17 expression, and treated cells display increased expression of the IL-23 receptor (R) [3]. This, together with the observation that IFN-γ decreases the expression of IL-23R in IFN-γ-deficient CD4^+ ^T cells differentiated towards a Th17 phenotype, indicates that IFN-γ is able to inhibit expression of the IL-23R. Additionally, IFN-γ-deficient mice have increased numbers of IL-17-producing T cells following mycobacterial infection as compared with wild-type mice, and exogenous IFN-γ reduces the frequency of IL-17-producing T cells in IFN-γ-deficient mice [9].

There is considerable evidence that IL-17 contributes to the inflammation associated with rheumatoid arthritis (RA). IL-17 is spontaneously produced by RA synovial membrane cultures and high levels of IL-17 were detected in the synovial fluid of patients with RA [10,11]. In collagen-induced arthritis (CIA), an animal model reminiscent in several aspects to RA, treatment with neutralizing anti-IL-17 antibody after the onset of arthritis reduces joint inflammation, cartilage destruction and bone erosion [12]. Authors proposed that the mechanisms responsible for slowing the disease are suppression of pro-inflammatory cytokines, such as IL-1β, TNF-α and IL-6, and elimination of the additive/synergistic effects between IL-17 and these pro-inflammatory cytokines. In addition, mice genetically deficient in IL-17 or IL-17R were found to be less susceptible for induction of CIA [13]. In contrast, local IL-17 overexpression accelerates the onset of CIA and aggravates synovial inflammation [14]. Evidence that IFN-γ regulates susceptibility to CIA through suppression of IL-17 comes from the observation that mice of the prototypical CIA-susceptible strain DBA/1 demonstrate a high IL-17 and low IFN-γ cytokine profile as compared with CIA-resistant C57BL/6 mice [15]. In addition, knocking out the IFN-γ gene renders the C57BL/6 mice susceptible to disease and switched their CD4^+ ^T cell differentiation towards Th17.

Despite the exciting new knowledge about Th17 cells and IL-17, their mechanisms of action in the pathogenesis of arthritis are still unclear. In the present study we investigated pro-inflammatory characteristics of IL-17 using the CIA model. As IFN-γ is counteracting the development of Th17 cells, we chose to induce CIA in IFN-γR knock out (KO) mice. Through neutralization of IL-17 using monoclonal anti-IL-17 antibody, we tested the effect of endogenous IL-17 on various potential effector targets of CIA, such as autoimmune cellular and humoral responses, production of cytokines and chemokines, stimulation of hematopoiesis and osteoclastogenesis. We found a clear-cut inhibition of CIA by treatment with anti-IL-17 antibody and the protection was associated with profound inhibition of myelopoiesis and production of myelopoietic cytokines. Extensive myelopoiesis is a well-described phenomenom in IFN-γR KO mice challenged with (auto)antigen in complete Freund's adjuvant (CFA; reviewed in [16,17]), so in an *in vitro *system using murine embryo fibroblast (MEF) cells we verified whether IL-17 may directly be involved in the induction of myelopoietic cytokines and/or chemokines and whether IFN-γ may influence this process.

## Materials and methods

### Antibodies and cytokines

Recombinant mouse IFN-γ was derived from the supernatant fluid of Mick cells, a Chinese hamster ovary (CHO) cell line developed in our laboratory [18]. IFN-γ was purified by affinity chromatography to a specific activity of 10^8.5^units/mg as described [19].

Anti-IL-17A antibody MM17F3 (IgG1-K) was derived from C57Bl/6 mice immunized with IL-17A-ovalbumin complexes as described [20]. Murine IgG1 monoclonal antibody (9E10), that recognizes part of the human c-*myc *protein, and is produced by the MYC1-9E10.2 (ATCC CRL 1729) hybridoma, was used as control antibody [21].

### Mice, induction, evaluation and treatment of CIA

Generation and basic characteristics of the mutant mouse strain (129/Sv/Ev) with a disruption in the gene encoding for the α-chain of the IFN-γ R (IFN-γR KO) have been described [22]. These IFN-γR KO mice were backcrossed with wild-type DBA/1 mice for 10 generations to obtain IFN-γR KO DBA/1 mice. Homozygous IFN-γR KO mice were identified by PCR as described [23]. Wild-type and IFN-γR KO DBA/1 mice were bred in the Experimental Animal Centre of the Rega Institute for Medical Research at Leuven.

The generation and basic characterization of IFN-γ-deficient mice of the 129 × BALB/c strain have been described [24]. These mice were backcrossed for eight generations to the parental BALB/c strain. The signal transducer and activator of transcription (STAT)-1^-/- ^and interferon regulatory factor (IRF)-1^-/- ^mice, on a C57BL/6 background, were from Dr. David E. Levy of the New York University School of Medicine (New York, USA) and from Dr. Tak Mak of the Ontario Cancer Institute (Ontario, Canada), respectively. The generation and characteristics of these mice have been described [25,26]. C57BL/6 mice (Harlan, Zeist, the Netherlands) were used as wild-type controls.

Chicken collagen type II (CII; Sigma-Aldrich, St Louis, MO, USA) was dissolved at 2 mg/ml in PBS containing 0.1 M acetic acid by stirring overnight at 6°C and emulsified in an equal volume of CFA (Difco Laboratories, Detroit, MI, USA) with added heat-killed *Mycobacterium butyricum *(1.5 mg/ml). Mice were sensitized with a single intradermal injection at the base of the tail with 100 μl of the emulsion on day 0, and treated with 0.2 mg of neutralizing anti-IL-17 or control antibody (in 250 μl PBS) once a week. Clinical and histological severity of arthritis were recorded following a scoring system as described [27,28].

All animal experiments were approved by the local ethical committee (University of Leuven).

### Measurement of total and anti-CII IgG antibodies and delayed-type hypersensitivity to CII

Blood samples were taken from the orbital sinus and were allowed to clot at room temperature for one hour and at 4°C overnight. Individual sera were tested for antibodies directed to chicken CII by ELISA as described [27]. For the determination of CII-specific IgG1, IgG2a and IgG2b antibody, plates were incubated for two hours with biotinylated rat antibody to mouse IgG1, IgG2a or IgG2b (Zymed Laboratories, San Francisco, CA, USA), followed by a one-hour incubation with streptavidin-conjugated peroxidase. For measurement of total IgG antibody, plates were coated with goat anti-mouse IgG (Jackson Immunoresearch Laboratories, West Grove, PA, USA; 10 μg/ml; 100 μl/well), followed by the same procedure as described in [27].

For evaluation of delayed-type hypersensitivity (DTH) reactivity, CII/CFA-immunized mice were subcutaneously injected with 20 μg of CII/20 μl PBS in the left ear and with 20 μl PBS in the right ear. DTH response was calculated as the percentage swelling (the difference between the increase of thickness of the left ear and the right ear, divided by the thickness of the right ear, multiplied by 100).

### Isolation of splenocytes and mouse embryo fibroblasts

Spleens were harvested, gently cut into small pieces and passed through cell strainers (Becton Dickinson Labware, Franklin Lakes, NJ, USA). Red blood cells were lysed by two consecutive incubations (5 and 3 minutes at 37°C) of the suspension in NH_4_Cl (0.83% in 0.01 M Tris-HCl, pH 7.2). Remaining cells were washed and counted.

To exclude any interference from endogenous IFN-γ, MEF were from IFN-γ KO BALB/c origin (unless otherwise mentioned). MEF were isolated from mouse embryos between 16 and 18 days of gestation, as described [28]. Two times 10^5 ^MEF in a total volume of 300 μl Eagle's minimal essential medium (EMEM) containing 2% heat-inactivated FCS were seeded in chamber slides (LAB-TEK Brand Products, Nalge Nunc International, Naperville, IL, USA). After an incubation of 48 hours, cells were stimulated with IL-17 (20 ng/ml) (R&D systems, Abingdon UK) and/or TNF-α (20 ng/ml) (R&D systems, Abingdon UK) in the presence or absence of IFN-γ (100 units/ml) for 48 hours. Supernatants were collected and cells were harvested using a cell scraper (LAB-TEK Brand Products, Nalge Nunc International, Naperville, IL, USA). Cells were washed and pellets were used for PCR.

### Flow cytometry

Single-cell splenocyte suspensions (0.5 × 10^6 ^cells) were incubated for 15 minutes with the Fc-receptor-blocking antibodies anti-CD16/anti-CD32 (BD Biosciences Pharmingen, San Diego, CA, USA). Cells were washed with PBS (2% FCS) and stained with the indicated fluorescein isothiocyanate (FITC)-conjugated antibodies (0.5 μg) for 30 minutes, washed and incubated with the indicated phycoerythrin (PE)-conjugated antibodies for 30 minutes. FITC-conjugated anti-CD25 (7D4) and FITC-conjugated anti-B220 were purchased from BD Biosciences Pharmingen, San Diego, CA, USA). FITC-conjugated anti-CD11b (M1/70), FITC-conjugated anti-CD8 (53-6.7), PE conjugated anti-CD4 (L3T4), and PE-conjugated anti-Gr-1 (RB6-8C5) were from eBioscience (ImmunoSource, Halle-Zoersel, Belgium). Cells were washed, fixed with 0.37% formaldehyde in PBS and flowcytometric analysis was performed on a FACScan flow cytometer with Cell Quest^® ^software (Becton Dickinson, San Jose, CA, USA).

### Detection of cytokines by quantitative PCR and ELISA

MEF were obtained as described above. RNA was extracted using the Micro-to-Midi Total RNA Purification System (Invitrogen Life Technologies, Carlsbad, CA, USA) in accordance with the manufacturer's instructions. cDNA was obtained by reverse transcription using Superscript II Reverse Transcriptase and random primers (Invitrogen Life Technologies, Carlsbad, CA, USA), in accordance with the manufacturer's instructions. For real-time PCR we used a TaqMan^® ^Assays-on-Demand™ Gene expression Product from Applied Biosystems (Foster City, CA, USA). Expression levels of granulocyte chemotactic protein-2 (GCP-2) (assay ID Mm00436451_g1, Applied Biosystems, Foster City, CA, USA), IL-6 (assay ID Mm00446190_m1, Applied Biosystems, Foster City, CA, USA), RANKL (assay ID Mm00441908_m1, Applied Biosystems, Foster City, CA, USA), IP-10 (assay ID Mm99999072_m1) and granulocyte macrophage colony-stimulating factor (GM-CSF; assay ID Mm99999059_m1) were normalized for 18S RNA (Cat. No. 4319413E, Applied Biosystems, Foster City, CA, USA) expression. Analysis was performed in an ABI Prism 7000 apparatus (Applied Biosystems, Foster City, CA, USA) under the following conditions: inactivation of possible contaminating amplicons by AmpErase uracil-N-glycosylase at 50°C for two minutes, initial denaturation at 95°C for 10 minutes, followed by 40 thermal cycles of 15 seconds at 95°C and 90 seconds at 60°C. The relative gene expression was assessed using the 2^-ΔΔCT ^method [29].

Expression of cytokines (i.e. IL-1β, IL-2, IL-4, IL-5, IL-6, IL-10, GM-CSF, IFN-γ and TNF-α) was determined by the Bio-Plex 200 system, Bio-plex Mouse Cytokine 8-plex assay and Bio-plex Mouse IL-6 Assay (Bio-Rad, Hercules, CA). IL-17, IP-10 and GM-CSF levels were measured by ELISA (R&D systems, Abingdon UK). GCP-2 was detected by an ELISA developed in our laboratory, as described [28].

## Results

### Increased levels of IL-17 in CII/CFA-immunized IFN-γR KO mice

**Figure 1:**
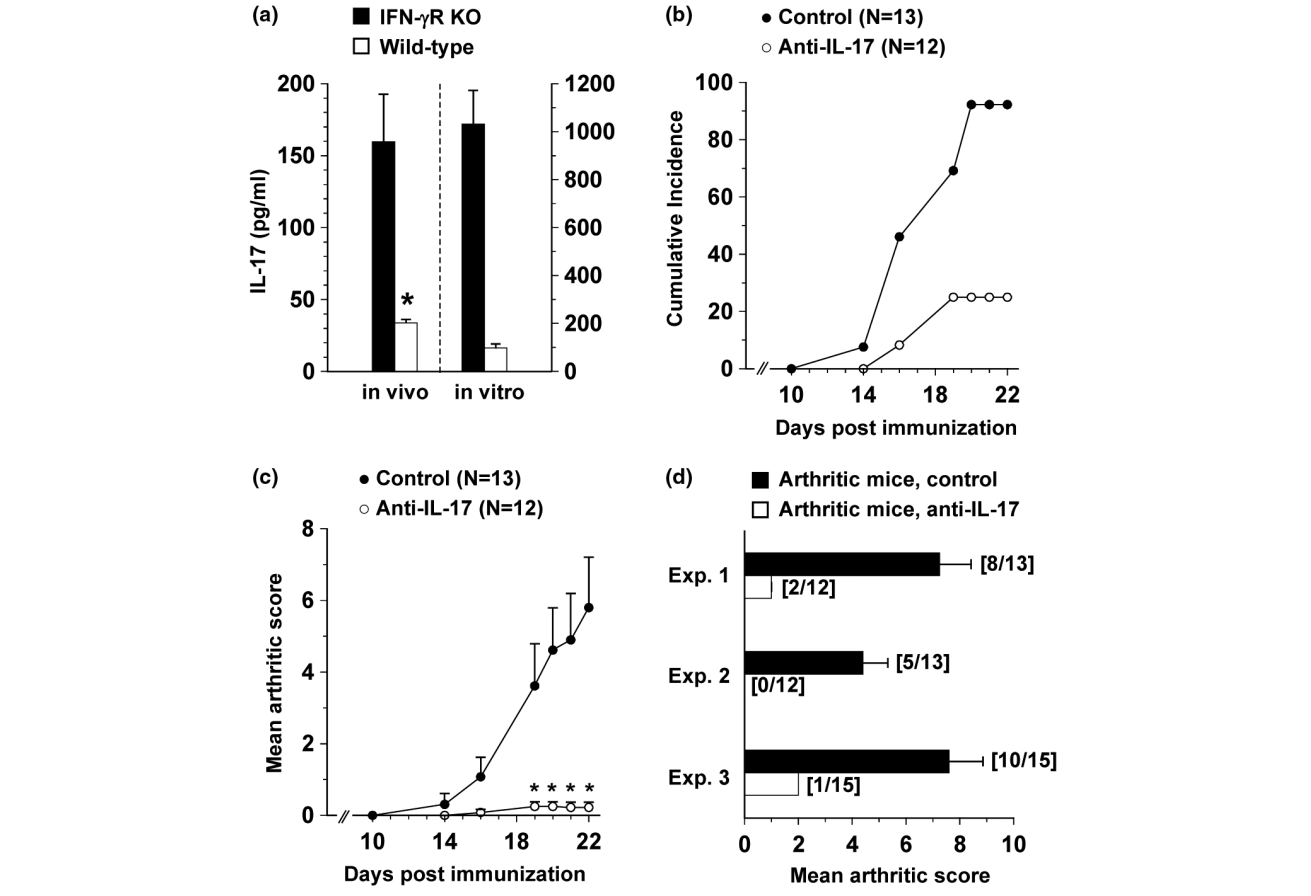
Treatment with anti-IL-17 antibody prevents CIA in IFN-γR KO mice. **(a) **Interferon-γ receptor knock-out (IFN-γR KO) and wild-type mice were immunized with collagen type II (CII) in complete Freund's adjuvant (*in vivo*). At day 21 post immunization, five IFN-γR KO and six wild-type mice were challenged with 10 μg anti-CD3 antibody and serum levels of IL-17 were determined 1.5 hours later. Bars represent averages ± standard error of the mean (SEM). *In vitro*: lymph nodes were isolated at day 35 post immunization, and lymphocytes were stimulated with 3 μg/ml anti-CD3 antibody. Supernatants were collected after 72 hours and tested for IL-17 expression. Bars represent averages ± SEM of triplicate cultures. * *P *< 0.05 for comparison with IFN-γR KO mice (Mann-Whitney U-test). **(b, c) **Immunized IFN-γR KO mice were injected with anti-IL-17 or control antibody. **(b) **Cumulative incidence of arthritis and **(c) **mean arthritic score of mice are shown. Error bars indicate SEM. Data are representative for three independent experiments. * *P *< 0.05 (day 19) and 0.005 (from day 20 onwards) for comparison with control-treated mice (Mann-Whitney U-test). **(d) **The mean arthritic score on day 21 of arthritic mice only is shown for three experiments. Error bars indicate SEM. The number of arthritic mice to the total number of mice in each group is shown in brackets.

IFN-γR KO mice develop CIA more readily than wild-type controls: symptoms of arthritis usually appear in IFN-γR KO mice from day 16 onwards compared with day 30 in wild-type animals [30]. In a first experiment, we confirmed the inhibitory activity of IFN-γ on the production of IL-17. Thus, IFN-γR KO and wild-type mice were immunized with CII in CFA. At day 21, a time point when the differences in disease symptoms between both groups are most pronounced, mice were injected with anti-CD3 and sera were collected. As expected, levels of IL-17 were more than four times higher in the sera of immunized IFN-γR KO mice as compared with those of wild-type counterparts (Figure 1a, *in vivo*). Similarly, anti-CD3-stimulated lymphocytes of immunized IFN-γR KO mice produced levels of IL-17 that were more than 10 times higher than those of wild-type mice (Figure 1a, *in vitro*). Therefore, to study the effector functions of IL-17 in arthritis, we chose IFN-γR KO mice for the induction of arthritis.

### Neutralization of IL-17 inhibits arthritis development in IFN-γR KO mice

**Figure 2:**
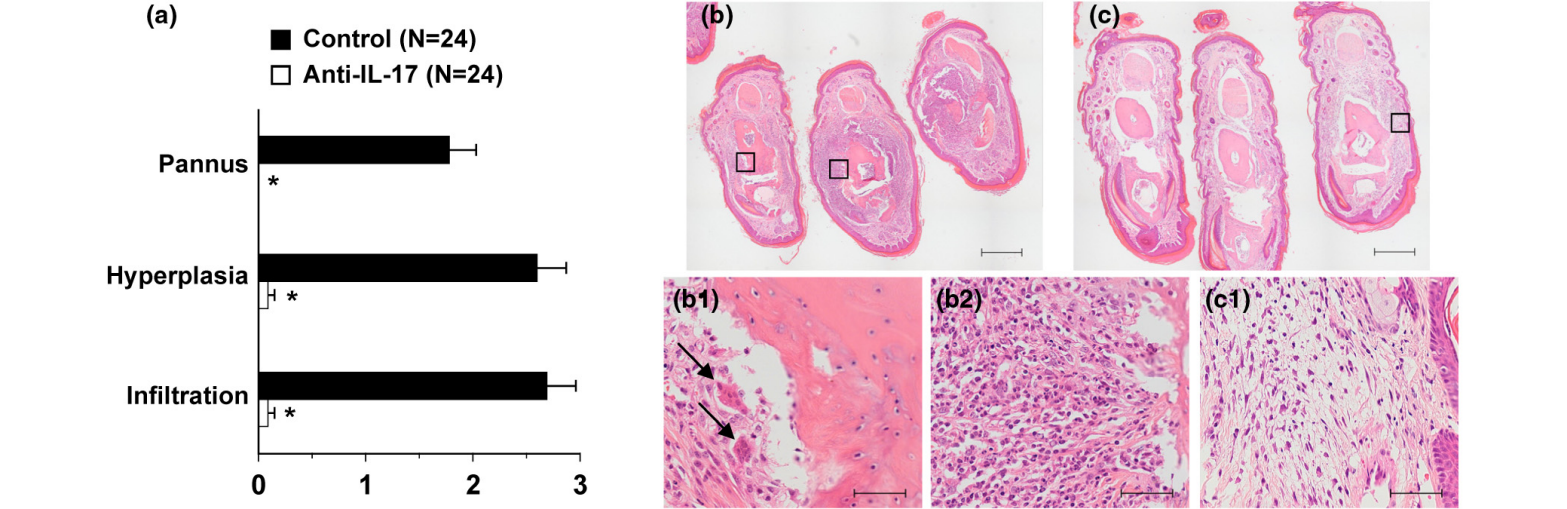
Absence of infiltration of neutrophils and bone destruction in the joints of anti-IL-17-treated mice. In experiment 1 of Figure 1d, histological analysis of the joints was performed. On day 25, four mice of each group were sacrificed and sections of the fore limbs, hind limbs and ankles were scored for three parameters of arthritis following hematoxylin staining. **(a) **Histograms represent averages ± standard error of the mean (SEM). * *P *< 0.005 for comparison with control-treated mice (Mann-Whitney U-test). **(b, c) **Pictures of the hematoxylin-stained paraffin sections of the joints of control-treated (**b**, a representative mouse) and anti-IL-17-treated (**c**, mice with the most severe symptoms) mice are shown. In control-treated mice, a severe hyperplasia and infiltration of immunocompetent cells in the synovium and pannus formation that penetrates into the bone can be seen. **(b1, 2) **Detail of the area indicated by respectively the left and right box in b. Note the presence of osteoclast-like multinucleated giant cells (arrows in b1), and the presence of polymorphonuclear cells in b2. **(c1) **Detail of the area indicated by the right box in c showing a moderate infiltration of mononuclear cells and hyperplasia. Scale bars represent **(b, c) **50 μm and **(b1, b2 and c1) **500 μm.

CII/CFA-immunized IFN-γR KO mice were injected with neutralizing anti-IL-17 or control antibody once a week starting from day 0 (day of immunization). In control-treated IFN-γR KO mice, symptoms of arthritis appeared from day 14 and reached a cumulative incidence of approximately 92%. In contrast, mice treated with anti-IL-17 antibody developed a significantly less severe form of arthritis with a lower incidence (25%) than control mice (Figures 1b, c). This protective effect was confirmed in two independent experiments. When the analysis was restricted to only arthritic mice, severity of arthritis was still lower in anti-IL-17-treated mice compared with control-treated mice (Figure 1d). In one such experiment, mice were sacrificed on day 25 for histological examination of the joints. As evident from data in Figure 2a, the reduced severity of arthritis in anti-IL-17-treated mice was associated with inhibition of infiltration of mono- and polymorphonuclear cells, hyperplasia and pannus formation (measured as the fraction of synovial inflammatory tissue, which has invaded bone tissue and forms bone erosion). In the control-treated group, several multinucleated osteoclast-like cells were detected at sites of bone erosion. Osteoclast-like cells and polymorphonuclear cells were completely absent in sections of anti-IL-17-treated mice. Figures 2b and 2c show light microscopy on hematoxylin-stained sections of both groups of mice.

### Reduced humoral and cellular auto-immune responses in IFN-γR KO mice treated with anti-IL-17 antibodies

**Figure 3:**
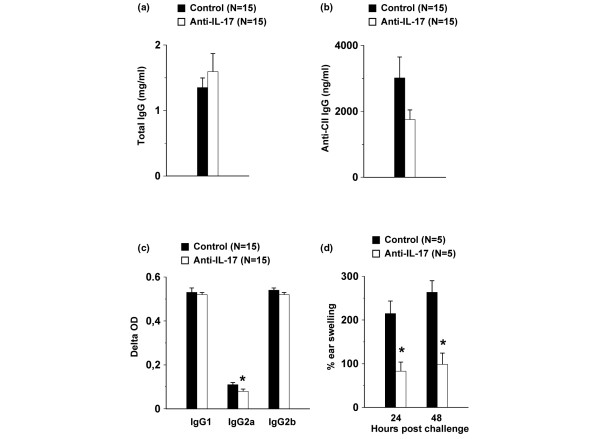
Reduced humoral and cellular immune response following treatment of CII/CFA immunized IFN-γR KO mice with anti-IL-17 antibodies. Interferon-γ receptor knock-out (IFN-γR KO) mice were immunized and injected intraperitoneally with 0.2 mg of neutralizing anti-IL-17 or control antibody once a week. **(a, b) **On day 22, sera of individual mice were analyzed for **(a) **total IgG, **(b) **anti- collagen type II (CII) IgG and **(c) **anti-CII IgG1, IgG2a and IgG2b. Histograms represent averages ± standard error of the mean (SEM). **(d) **25 days after immunization, mice in each group were challenged with 20 μg of CII in the left ear and vehicle in the right ear. Delayed type hypersensitivity responses were measured as the percentage of swelling (i.e. 100 × the difference between the increase of thickness of the left and the right ears, divided by the thickness of the right ear) at the indicated time points. Histograms represent averages ± SEM. * *P *< 0.05 for comparison with control-treated mice (Mann-Whitney U-test). CFA = complete Freund's adjuvant.

Pathogenesis of CIA is generally considered to depend in part on both humoral and cellular immune responses against CII. We wanted to define whether the protection against CIA by anti-IL-17 antibodies results from modulation from either of these. Total IgG and anti-CII IgG, IgG1, IgG2a and IgG2b were determined in the sera of anti-IL-17- and control-treated IFN-γR KO mice on day 22 post-immunization. No differences in total IgG serum content were detected in the sera of anti-IL-17- and control-treated animals (Figure 3a). Titers of anti-CII antibodies were found to be reduced in the sera of anti-IL-17-treated mice, although values did not reach statistical significance (Figure 3b). As to subtypes of anti-CII IgG, no differences in anti-CII IgG1 and IgG2b could be detected. However, levels of anti-CII IgG2a were significantly lower in sera of anti-IL-17-treated IFN-γR KO mice as compared with control-treated mice (Figure 3c). DTH was tested on day 25 after immunization by injecting 20 μg of CII in the left ears and vehicle in the right ears. Bars in Figure 3d represent the percentages of swelling in the CII-challenged ears, normalized to the swelling of the PBS-challenged ears. A significantly lower DTH response to CII was observed in the anti-IL-17-treated mice as compared with that in control mice. Thus, the reduced severity of arthritis in anti-IL-17-treated mice appeared to be associated with reduced cellular immune responsiveness to CII.

### Inhibition of arthritis in IFN-γR KO mice is associated with reduced expansion of CD11b+ cells

**Figure 4:**
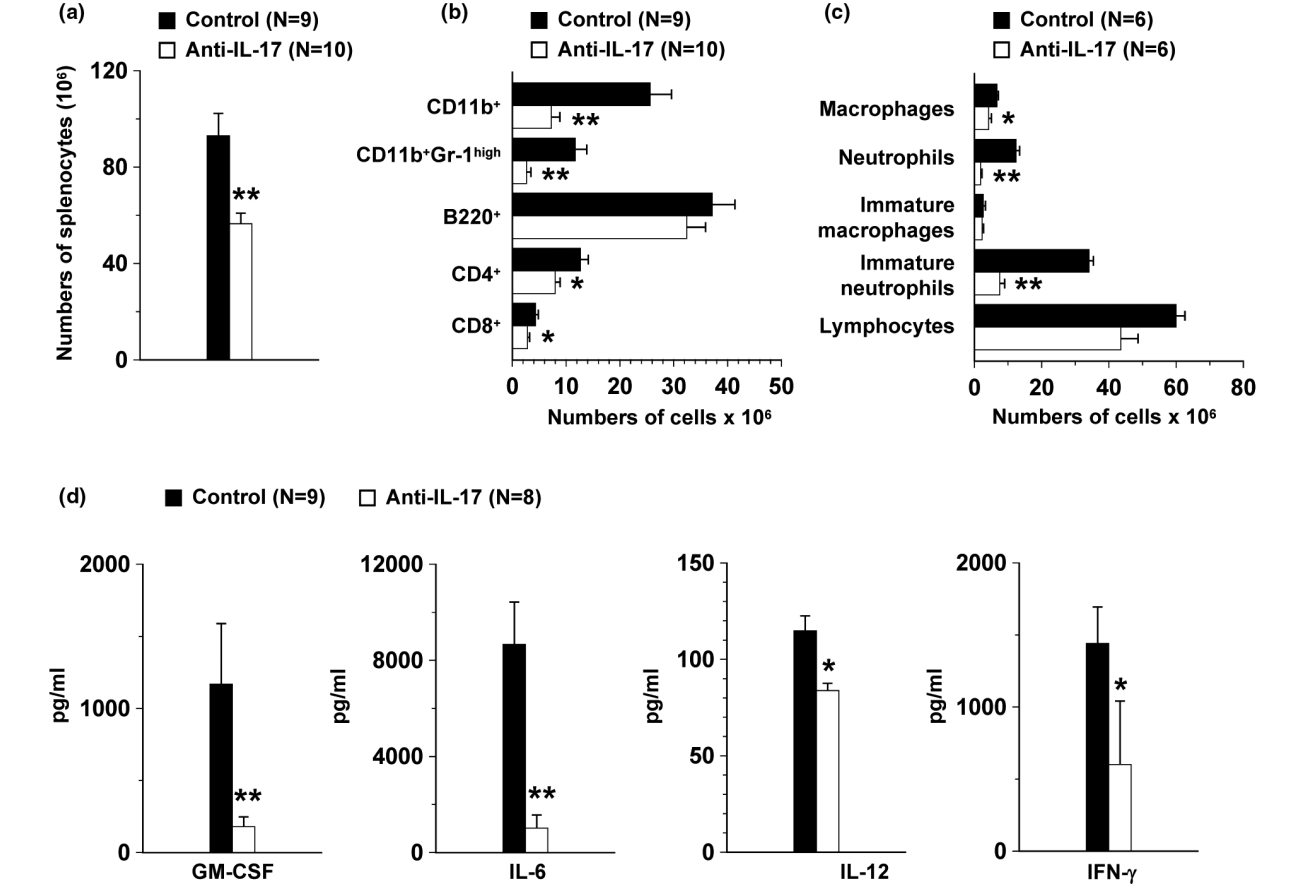
Anti-IL-17 antibody treatment reduces the splenic CD11b^+ ^cell expansion and the systemic production of myelopoietic cytokines. Interferon-γ receptor knock-out (IFN-γR KO) mice were immunized and injected intraperitoneally with anti-IL-17 or control antibody once a week. **(a to c) **On day 21, spleens of individual mice were isolated and splenocytes were counted. **(a) **Bars represent the mean number of splenocytes ± standard error of the mean (SEM). **(b) **Splenocytes were characterized by flow cytometry and the percentage of CD11b^+ ^cells, CD11b^+^Gr-1^high ^cells, B220^+ ^cells, CD4^+ ^cells and CD8^+ ^cells was analyzed. Bars represent the mean net numbers of three independent experiments, each consisting of three to four mice per group ± SEM. **(c) **The percentage of lymphocytes, (immature) neutrophils and macrophages was assessed on cytospin splenocyte preparations of six anti-IL-17- and six control-treated mice, and the net numbers were calculated. Bars represent net numbers ± SEM. **(d) **At day 21 post immunization, mice were challenged with 10 μg anti-CD3 antibody and serum levels of granulocyte macrophage colony-stimulating factor (GM-CSF), IL-6, IL-12 and IFN-γ were determined 1.5 hours later. Bars represent the mean of two independent experiments, each consisting of four to five mice per group ± SEM. * *P *< 0.05 and ** *P *< 0.005 for comparison with control-treated mice (Mann-Whitney U-test).

The more severe form of arthritis in IFN-γR KO mice as compared with wild-type mice is accompanied with an extramedullary hemopoiesis and expansion of the CD11b^+ ^cell population [31]. These expanding CD11b^+ ^splenocytes, containing mostly immature mononuclear phagocytes and neutrophils, can act as a source of osteoclasts and may thus indirectly account for bone destruction in CIA [23] or may contribute to neutrophil inflammation in the joints. IL-17 is known to stimulate granulopoiesis *in vivo *[32] and to induce the production of hematopoietic cytokines such as IL-6, IL-8 and G-CSF *in vitro *[5], so we analysed the effect of IL-17 neutralization on the expansion of the CD11b^+ ^cell expansion. Therefore, spleens were isolated from anti-IL-17-treated and control-treated IFN-γR KO mice on day 21 after immunization. The mean number of splenocytes was significantly lower in mice treated with anti-IL-17 as compared with control antibody (Figure 4a). To characterize these splenocytes, flowcytometric analysis was performed. Figure 4b shows that the anti-IL-17 antibodies inhibit the expansion of the CD11b^+ ^cell population. In fact, treatment with anti-IL-17 antibodies resulted in significantly lower net numbers of CD11b^+^Gr-1^high ^neutrophils in the spleen. Numbers of CD4^+ ^and CD8^+ ^T cells were also significantly lower in anti-IL-17-treated mice, although to a lesser degree. B220^+ ^cell numbers were comparable in both groups of mice. These data were confirmed by cytospin splenocyte preparations (Figure 4c). Significantly lower numbers of immature and mature neutrophils were observed in the spleen of anti-IL-17-treated as compared with control-treated mice. Although to a lesser degree, a significant reduction in the number of macrophages in the splenocyte population of anti-IL-17-treated mice was also seen. Treatment with anti-IL-17 antibodies did not significantly affect the number of lymphocytes in the splenocyte preparations.

### Anti-IL-17 treatment in IFN-γR KO mice decreases the production of hemopoietic cytokines

The reduced expansion of the CD11b^+ ^cell population following treatment with anti-IL-17 antibodies might result from inhibition of the production of hemopoietic cytokines. Therefore, sera of anti-CD3-challenged IFN-γR KO mice treated with anti-IL-17 or control antibodies were collected on day 21 and tested for the presence of cytokines. Levels of GM-CSF, IL-6 and IL-12, known to stimulate hemopoiesis, were significantly reduced in the sera of mice treated with anti-IL-17 antibodies as compared with control-treated mice (Figure 4d), possibly providing an explanation for the reduced expansion of CD11b^+ ^cells in the spleen of anti-IL-17-treated mice. Expression of IFN-γ, known to inhibit the CFA-induced expansion of CD11b^+ ^cells, was also found to be significantly reduced upon treatment with anti-IL-17. However, as IFN-γR KO mice were used, levels of IFN-γ have no effect in our model. With regard to the expression of other cytokines, levels of the pro-inflammatory cytokine TNF-α were slightly reduced upon treatment with anti-IL-17 antibodies (1133.3 ± 658.6 and 721.9 ± 562.3 pg/ml for the control- and anti-IL-17-treated mice, respectively), and expression of all other tested cytokines (IL-2, IL-4, IL-5 and IL-10) were comparable in the sera of both groups of mice (data not shown).

### IL-17 induces the production of IL-6, RANKL and GCP-2 in mouse embryo fibroblasts, and this induction is potently inhibited by IFN-γ

**Figure 5:**
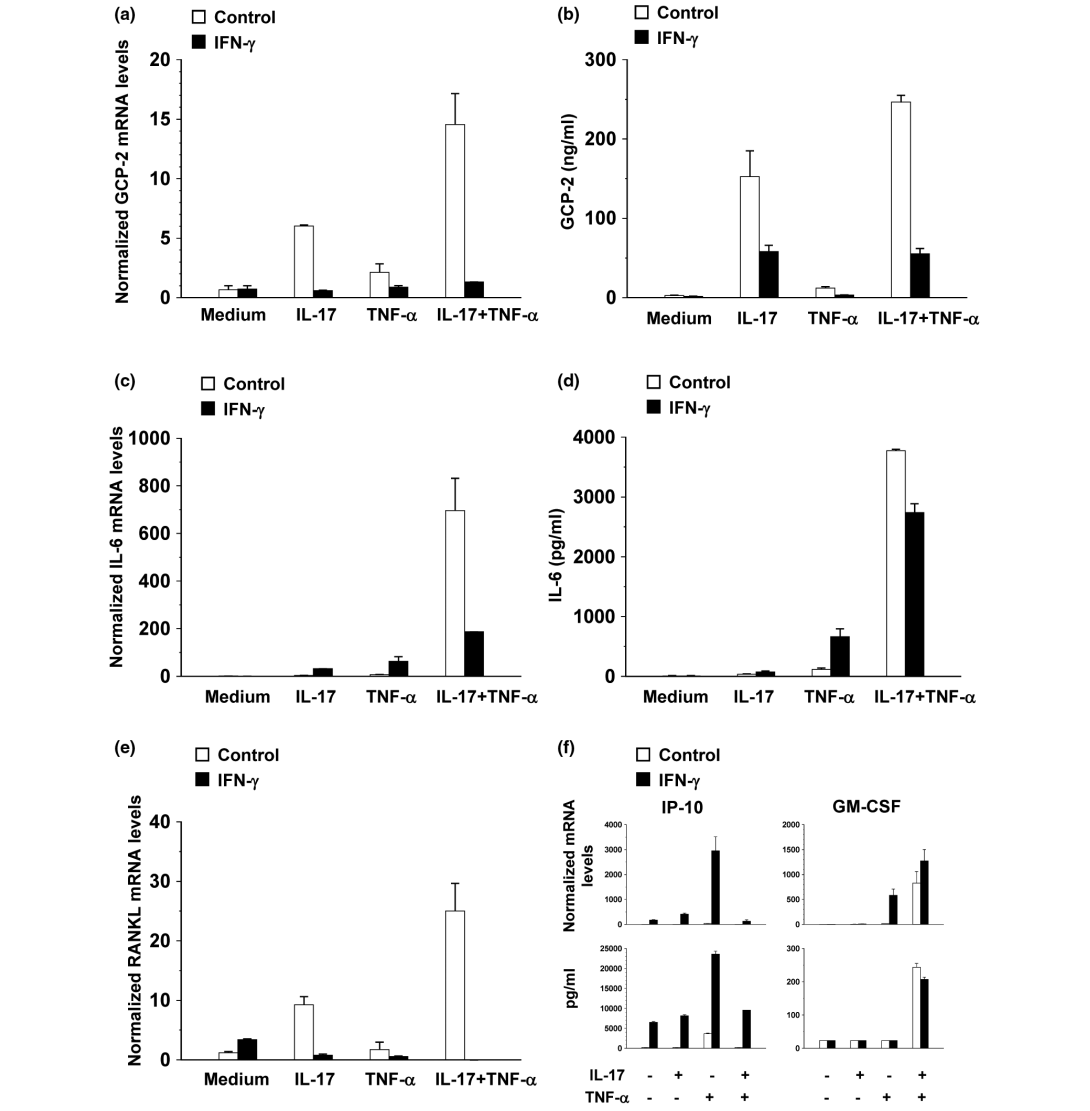
Synergy of IL-17 and TNF-α in the induction of GCP-2, RANKL and IL-6 and inhibition by IFN-γ. **(a to d) **Mouse embryo fibroblasts (MEF) cells of interferon-γ ligand knock-out (IFN-γ ligand KO) BALB/c mice were grown to confluence and stimulated for 48 hours with IL-17 (20 ng/ml) and/or TNF-α (20 ng/ml), or were left untreated in the absence or presence of IFN-γ (100 units/ml). **(a, c, e and f) **cDNA samples were prepared and subjected to quantitative PCR analysis. The relative quantity of granulocyte chemotactic protein-2 (GCP-2), receptor activator of nuclear factor-κB ligand (RANKL), IL-6, IP-10 and granulocyte macrophage colony-stimulating factor (GM-CSF) mRNA in each sample was normalized to the quantity of 18S RNA. **(b, f) **GCP-2, interferon-gamma-induced protein (IP-10) and GM-CSF protein present in the supernatants of stimulated MEF was measured by ELISA. **(d) **IL-6 protein present in the supernatants was quantified by the Bioplex system. Results represent the mean of two cultures ± standard error of the mean. Results are representative for **(a, b) **four and **(c to f) **two independent experiments.

In a next set of experiments, we verified whether the absence of bone destruction and reduced influx of neutrophils in the joints, as well as the reduced production of hematopoietic cytokines such as IL-6 upon anti-IL-17-antibody treatment, is indirectly due to the reduced inflammation seen in these mice or may directly result from neutralization of IL-17. As to the inhibition of bone destruction, we compared the osteoclastogenic capacity of splenocyte populations of immunized anti-IL-17-treated and control-treated mice. Thus, splenocytes from both groups of mice were stimulated with macrophage colony-stimulating factor (M-CSF) and RANKL for induction of osteoclastogenesis. We found similar numbers and activity of osteoclasts in both groups of mice (data not shown), indicating that osteoclast precursors are present in both splenocyte populations. Possibly, the migration of these osteoclast precursors to the joints or the production of osteoclast stimulating factors such as RANKL in the joints is disturbed in anti-IL-17-treated mice. As fibroblasts are an important source of RANKL, IL-6 and the neutrophil-specific chemokine GCP-2 ([28]and our unpublished results), and because of the limited source of mouse synovial fibroblasts, we chose to use MEF for *in vitro *stimulation. Cells were stimulated with IL-17 in the absence or presence of TNF-α for 48 hours, and mRNA levels were measured using quantitative PCR. In addition, we tested the effect of IFN-γ on the induction of RANKL, IL-6 and GCP-2. As shown in Figure 5a, IL-17 induced the expression of GCP-2 mRNA in MEF. Moreover, a synergy between IL-17 and TNF-α in the induction of GCP-2 mRNA could be observed. These findings were confirmed at the protein level, using a GCP-2-specific ELISA (Figure 5b). IL-17 and TNF-α were also found to synergistically induce the expression of IL-6 mRNA (Figure 5c), IL-6 protein (Figure 5d) and RANKL mRNA (Figure 5e). These data indicate that IL-17 is able to induce the production of neutrophil-specific chemokines such as GCP-2. Through induction of IL-6 and RANKL, IL-17 can stimulate hemopoiesis and bone destruction. Importantly, expression of GCP-2, IL-6 and RANKL was counteracted by IFN-γ (Figures 5a to 5e). Thus, aside from inhibition of the production of IL-17, IFN-γ can inhibit the effector function of IL-17. To exclude that IFN-γ non-specifically inhibits cytokine production, we also tested the effect of IFN-γ on the production of keratinocyte-derived chemokine (KC), macrophage inflammatory protein (MIP)-2, IP-10, Regulated upon Activation, Normal T-cell Expressed, and Secreted (RANTES), monocyte chemotactic protein (MCP)-1, interferon-inducible T cell alpha chemoattractant (ITAC), and monokine induced by gamma interferon (MIG). IFN-γ was found to exhibit stimulatory effects on the production of MIP-2, IP-10, RANTES, ITAC and MIG induced by IL-17 and/or TNF-α (Figure 5f and data not shown). No significant effect of IFN-γ was observed on the production of M-CSF, KC and MCP-1 (Figure 5f and data not shown).

### Inhibition of the effector function of IL-17 by IFN-γ is STAT-1- but not IRF-1-dependent

**Figure 6:**
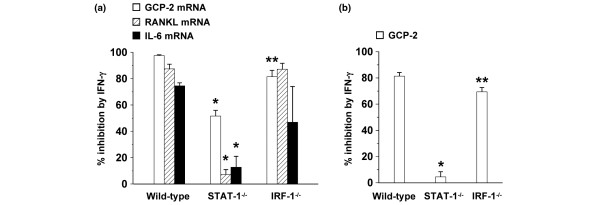
Inhibition of effector functions of IL-17 by IFN-γ is STAT-1-dependent. Mouse embryo fibroblasts (MEF) of wild-type, signal transducer and activator of transcription (STAT)-1^-/- ^and interferon regulatory factor (IRF)-1^-/- ^C57BL/6 mice were grown to confluence and stimulated for 48 hours with IL-17 (20 ng/ml) and TNF-α (20 ng/ml), or were left untreated in the absence or presence of IFN-γ (100 units/ml). **(a) **cDNA samples were prepared and subjected to quantitative PCR analysis. The relative quantity of granulocyte chemotactic protein-2 (GCP-2), receptor activator of nuclear factor-κB ligand (RANKL) and IL-6 mRNA in each sample was normalized to the quantity of 18S RNA. **(b) **GCP-2 protein present in the supernatants of stimulated MEF was measured by ELISA.**(a, b) **All cultures were performed in duplicate/triplicate in two independent experiments and the percentage of inhibition was calculated as 100 × (expression in condition without IFN-γ – expression in condition with IFNγ)/expression in condition without IFN-γ). Results represent the mean of five cultures ± standard error of the mean. * *P *< 0.05 for comparison with corresponding wild-type and, except for IL-6, IRF-1^-/- ^condition and ** *P *< 0.05 for comparison with corresponding wild-type and STAT-1^-/- ^condition.

STAT-1 is a major mediator of cell activation by IFN-γ [33]. However, IFN-γ also activates STAT-1-independent pathways, and it has been proposed that these STAT-1-independent pathways mediate suppressive activities of IFN-γ. To investigate whether suppression of GCP-2, IL-6 and RANKL expression by IFN-γ was STAT-1-dependent, MEF from wild-type and STAT-1^-/- ^mice of the C57BL/6 strain were prepared. At the same time, we investigated whether the inhibition by IFN-γ was dependent on IRF-1, a transcription factor acting immediately downstream of STAT-1, by preparing MEF from IRF-1^-/- ^C57BL/6 mice. In each of the MEF, IL-17 and TNF-α synergistically induced the expression of GCP-2, RANKL and IL-6 mRNA (data not shown). To evaluate the importance of STAT-1 and IRF-1 in the inhibitory action of IFN-γ, the percentage of inhibition of the expression of GCP-2, RANKL and IL-6 mRNA induced by synergistic action of IL-17 and TNF-α was calculated and compared. As shown in Figure 6a, the inhibition by IFN-γ was significantly reduced in STAT-1^-/-^, but unaffected in IRF-1^-/- ^MEF, as compared with wild-type MEF. For the inhibition of the expression of GCP-2, these results were confirmed at the protein level (Figure 6b). These data indicate that the inhibition by IFN-γ is STAT-1-, but not IRF-1-dependent.

## Discussion

IL-17 plays a key inflammatory role in the propagation of RA and CIA [11,34,35]. IL-17 promotes inflammation through enhancing the production of inflammatory cytokines, such as IL-1β, TNF-α and RANKL, as well as neutrophil-specific chemokines such as MIP-2 and IL-8 [4,5,36]. Inhibition of the IFN-γ signaling enhances development of pathogenic Th17 cells that can exacerbate autoimmunity [3,9]. IFN-γR KO mice have been found to experience an accelerated and more severe form of CIA [30]. Events contributing to this protective effect of IFN-γ in CIA are inhibition of the CFA-induced myelopoiesis and osteoclastogenesis, inhibition of the production of GCP-2 and thus neutrophil infiltration, and stimulation of T_reg _cell activity [23,27,28,31]. Recently, IFN-γ was found to regulate susceptibility to arthritis through suppression of IL-17 [15,37]. Although Th17 cells and IL-17 have been studied extensively in previous years, much of their effector functions remain unknown. To observe maximal effects of IL-17 neutralization in CIA, we chose to start from IFN-γR KO mice for the induction of CIA. A preventive treatment with the anti-IL-17 antibodies (starting from the day of the immunization), almost completely abrogated arthritis development in IFN-γR KO mice and inhibited the influx of immunocompetent cells (predominantly neutrophils), hyperplasia of the synovial membrane and bone destruction. Lubberts and colleagues found that treatment with anti-IL-17 antibody after the onset of CIA significantly reduces the severity of CIA in wild-type mice [12]. In line with our results, lower numbers of multinucleated cells were present in the joints of the anti-IL-17-treated mice as compared with control mice.

IL-17 is known to be a potent stimulator of osteoclastogenesis through induction of RANKL [10,38]. Nonetheless, if splenocytes from immunized anti-IL-17-treated and control mice were stimulated *ex vivo *with RANKL and M-CSF for induction of osteoclastogenesis, in the absence or presence of exogenously added neutralizing anti-IL-17 antibody, no differences could be observed in the numbers or activity of osteoclasts (data not shown). More recently, however, Sato and colleagues have demonstrated that IL-17 has no effect on osteoclastogenesis in the RANKL-M-CSF system, but promotes osteoclast differentiation in the co-culture system through the induction of RANKL on osteoblastic cells [39].

Joint inflammation in CIA is assumed to depend in part on collagen-specific humoral and cellular immune reactivity. Treatment with anti-IL-17 antibody resulted in reduced, although not significantly, titers of total anti-CII IgG antibodies. No differences in anti-CII IgG1 and IgG2b could be detected, but significantly lower levels of anti-CII IgG2a in sera of anti-IL-17-treated IFN-γR KO mice were established. As IgG2a and IgG1 kinetics indirectly reflect Th1/Th2 responses, these data are suggestive for a lower Th1 response after treatment with anti-IL-17 antibodies. IL-17 was previously shown to be important in the induction of autoreactive humoral immune responses because a deficiency in this cytokine is associated with a decline in the autoantibody response in CIA and experimental autoimmune encephalomyelitis [13,40]. Recently, IL-17 was found to drive autoimmune responses by promoting the formation of spontaneous germinal centers [41]. With regard to the cellular immune responses, we found significantly impaired DTH to CII in IFN-γR KO mice treated with anti-IL-17 antibody. In IL-17 KO mice, Nakae and colleagues have established significantly reduced proliferative responses of lymph node cells against CII in comparison with that of wild-type mice [13]. Taken together, these results demonstrate a crucial role for IL-17 in the activation of CII-specific cellular responses.

As we reported earlier [31], the increased severity of CIA in IFN-γR KO mice is associated with an increased CFA-induced extramedullary expansion of immature CD11b^+ ^macrophages and neutrophils. In the present study we showed that anti-IL-17 antibodies inhibit the expansion of this CD11b^+ ^cell population. Characterization of these CD11b^+ ^splenocytes indicated a significant reduction in the numbers of (immature) neutrophils. The lower numbers of neutrophils present in the spleen may provide an explanation for the reduced influx of neutrophils in the joints of the anti-IL-17-treated mice. IL-17 has been found to act as a stimulatory hematopoietic cytokine by expanding myeloid progenitors and initiating proliferation of mature neutrophils [42]. It has been described to induce the secretion of hematopoietic cytokines such as IL-6 and G-CSF in fibroblasts, through which these stimulated fibroblasts can sustain the proliferation of hematopoietic progenitors and their preferential maturation into neutrophils [5]. In the same line, IL-17R KO mice have impaired hemopoietic recovery following gamma irradiation; hemopoietic precursors are reduced by 50% and neutrophils by 43% [43]. In the present study, the reduced granulopoiesis observed in anti-IL-17-treated mice probably resulted from reduced production of GM-CSF, IL-6 and IL-12. Treatment with neutralizing anti-IL-17 antibodies after the onset of arthritis has also been demonstrated to result in significantly reduced systemic IL-6 levels in wild-type mice [12]. Along the same line, stimulation of various cell types with IL-17 has been found to induce production of pro-inflammatory mediators such as IL-6 and GM-CSF [4,44,45].

In a previous set of experiments we tested whether the observed reduced influx of neutrophils in the joints, bone destruction and hematopoiesis directly result from neutralization of IL-17, or were a reflexion of the reduced inflammation present in the anti-IL-17-treated mice. In mice, IL-8, the prototype of human CXC chemokines that mainly affect neutrophil migration, has not been described, but the only neutrophil-attracting chemokine recognizing CXCR1 and 2 is GCP-2, generally considered as the functional equivalent of IL-8 in humans [46,47]. We found that IL-17 and TNF-α synergistically induce the production of GCP-2, RANKL, IL-6 and GM-CSF in MEF, thereby providing an explanation for the observed phenomena upon neutralization of IL-17. The induction of IL-6 and RANKL by IL-17 has been described [4,48,49]. In addition, studies have already linked IL-17 to induction of neutrophil-specific chemokines such as IL-8 and MIP-2 and the consequent effects on neutrophil recruitment [5,6,49]. More recently, IL-17 was shown to promote the selective expression of ELR^+ ^CXC chemokines in RA synoviocytes, especially in the presence of TNF-α, highlighting its role in neutrophil recruitment into the joint [49]. Furthermore, IL-17 induces the production of GCP-2 in a preosteoblast cell line and normal mesenchymal cells [50,51]. Shen and colleagues have shown that the CXC family chemokines, including KC, MIP-2 and GCP-2, were dramatically induced by IL-17 and/or TNF-α [48]. Recently, IL-17 was found to enhance cartilage destruction by increasing the production of KC and MIP-2, and thereby the influx of Fcγ R-bearing neutrophils [52]. Importantly, we established an inhibition of this production of GCP-2, RANKL and IL-6 upon addition of IFN-γ. Levels of other tested cytokines induced by TNF-α/IL-17 were upregulated or not affected by IFN-γ, indicating that inhibition was specific. Although IFN-γ has been described to regulate neutrophil influx through inhibition of the IL-1β-, TNF-α-driven or mycobacteria-driven production of IL-8 and/or GCP-2 [28,51,53,54], its effect on IL-17-induced gene expression was so far not investigated. Using STAT-1^-/- ^and IRF-1^-/- ^MEF, we found that the observed inhibition by IFN-γ is IRF-1-independent, but STAT-1-dependent. Taken together, aside from the well-known inhibition of the development of Th17 cells by IFN-γ, we demonstrate that IFN-γ profoundly inhibits the effector function of IL-17 in a STAT-1-dependent way.

As levels of IL-17 were found to be significantly lower in wild-type mice as compared with IFN-γR KO mice and because IFN-γ profoundly inhibits the effector function of IL-17, we expected the protective effect of anti-IL-17 in wild-type mice to be less pronounced. However, a preventive anti-IL-17 antibody treatment significantly inhibited the clinical and histological symptoms of arthritis in wild-type mice (with a total of 16 mice in each group, an incidence was reached of 54% and 13% respectively in control-treated and anti-IL-17-treated wild-type mice). In an attempt to unravel the mechanism of protection, we analysed the effect of anti-IL-17 treatment on different effector targets. Whereas the CII cellular autoimmune response (i.e. DTH) was found to be reduced by half upon treatment with anti-IL-17 antibody, the production of anti-CII autoantibodies and the splenic expansion of CD11b^+ ^cells were not affected (data not shown). Thus, although anti-IL-17 antibody is effective in reducing the clinical symptoms of arthritis in both IFN-γR KO and wild-type mice, the mechanism of endogenous IL-17 in the pathogenesis of CIA appears to be somewhat different between the two groups of mice.

It is generally accepted that different pathways, such as humoral and cellular immune responses, myelopoiesis and osteoclastogenesis are all required to develop CIA. Depending on the presence or absence of IFN-γ, IL-17 mediated several of these processes and it can be speculated that blocking just one of these pathways, for example DTH, may be sufficient to prevent symptoms of arthritis. Alternatively, there may exist a profound effector mechanism of IL-17 in both IFN-γR KO and wild-type mice that has not been investigated in our study and still needs to be discovered.

## Conclusions

In conclusion, our experiments underscore that IL-17 mediates its pro-inflammatory role in CIA in IFN-γR KO mice mainly through stimulatory effects on granulopoiesis, neutrophil infiltration and bone destruction. Importantly, our data reveal also an additional mechanism through which IFN-γ can attenuate some autoimmune diseases and autoimmune arthritis in particular. Apart from the inhibition of the production of IL-17, IFN-γ also abrogates some of the effector functions of IL-17. Thus, through its inhibition of the IL-17-induced production of IL-6, GCP-2 and RANKL, IFN-γ can profoundly limit granulopoiesis, mobilisation of neutrophils, and bone destruction, which are all important in joint inflammation.

## Abbreviations

CFA: complete Freund's adjuvant; CHO: Chinese hamster ovary; CIA: collagen-induced arthritis; CII: collagen type II; DTH: delayed type hypersensitivity; ELISA: enzyme-linked immunosorbent assay; EMEM: Eagle's minimal essential medium; FCS: fetal calf serum; FITC: fluorescein isothiocyanate; GCP-2: granulocyte chemotactic protein-2; G-CSF: granulocyte colony-stimulating factor; GM-CSF: granulocyte macrophage colony-stimulating factor; IFN: interferon; IFN-γR KO: interferon-γ receptor knock-out; IL: interleukin; IP-10: interferon-gamma-induced protein; IRF: interferon regulatory factor; ITAC: interferon-inducible T cell alpha chemoattractant; KC: keratinocyte-derived chemokine; MCP: monocyte chemotactic protein; M-CSF: macrophage colony-stimulating factor; MEF: mouse embryo fibroblasts; MIG: monokine induced by gamma interferon; MIP: macrophage inflammatory protein; PBS: phosphate buffered saline; PCR: polymerase chain reaction; PE: phycoerythrin; RA: rheumatoid arthritis; RANKL: receptor activator of nuclear factor-κB ligand; RANTES: Regulated upon Activation, Normal T-cell Expressed, and Secreted; STAT: signal transducer and activator of transcription; TGF-β: transforming growth factor-β; TNF-α: tumor necrosis factor-α.

## Competing interests

The authors declare that they have no competing interests.

## Authors' contributions

CU and JVS prepared and evaluated anti-IL-17 antibody. HK, ES and LG induced and evaluated CIA. PM, HK and ES measured histology, humoral and cellular responses. HK and TM measured flow cytometry and cytospins. HK, ES, TM, LG and JVD conducted MEF stimulations, ELISA, quantitative PCR and Bioplex. PM, HK and JVS designed the study. All authors were involved in interpreting the data. HK, PM, JVD and JVS prepared the manuscript.
